# *Campylobacter fetus* Bacteremia Related to Vascular Prosthesis and Pseudoaneurysm Infection: A Case Report and Review

**DOI:** 10.3390/pathogens11121536

**Published:** 2022-12-14

**Authors:** Karolina Dobrović, Branko Fila, Andrea Janeš, Rok Civljak

**Affiliations:** 1 Department of Clinical Microbiology and Hospital Infections, Dubrava University Hospital, 10000 Zagreb, Croatia; 2 Department of Vascular Surgery, Dubrava University Hospital, 10000 Zagreb, Croatia; 3 Department of Clinical Microbiology and Hospital Infections, University Hospital “Sveti Duh”, 10000 Zagreb, Croatia; 4 Department for Acute Respiratory Tract Infections, University Hospital for Infectious Diseases “Dr. Fran Mihaljević”, 10000 Zagreb, Croatia; 5 Department of Infectious Diseases, University of Zagreb School of Medicine, 10000 Zagreb, Croatia

**Keywords:** bacteremia, *Campylobacter fetus*, infection, pseudoaneurysm, vascular prosthesis

## Abstract

Background. *Campylobacter fetus* rarely causes gastrointestinal diseases but shows an affinity for the endovascular epithelium. Methods. We describe a case of *C. fetus* bacteremia related to vascular prosthesis and pseudoaneurysm infection, with a review of the literature. Results. A 67-year-old male was admitted with a history of fever, weakness and painful swelling of the groin. After unsuccessful treatment with ciprofloxacin, the patient was transferred to our hospital, where he had been previously treated for aortoiliac occlusive disease including a prosthetic aortobifemoral and popliteal bypass with polyester graft placement. An angiography showed a pseudoaneurysm in the groin and, therefore, repair of the pseudoaneurysm, removal of the prosthesis and biologic graft placement were performed. Blood cultures and tissue samples of the vascular prosthesis and pseudoaneurysm yielded *C. fetus* resistant to ciprofloxacin. The patient was treated with meropenem for four weeks, followed by amoxicillin-clavulanate for another two weeks after discharge. Eight previously published cases of *C. fetus* bacteremia due to infected cardiovascular prosthetic devices (prosthetic heart valves, implantable cardioverter-defibrillators and a permanent pacemaker) were summarized in the review. Conclusions. To our knowledge, this is the first report of a *C. fetus* bacteremia related to post-surgical infection of a vascular prosthesis causing a pseudoaneurysm.

## 1. Introduction

In humans, *Campylobacter* spp. have been associated with a range of gastrointestinal conditions, including inflammatory bowel diseases (IBD), Barrett’s esophagus and colorectal cancer. Diarrheal disease due to *Campylobacter* spp. is mainly caused by *Campylobacter jejuni* and *Campylobacter coli* [1]. On the other hand, *Campylobacter fetus* rarely causes gastrointestinal disease but can lead to blood dissemination, with signs and symptoms of the seeding organ. *Campylobacter fetus* exhibits an affinity for the endovascular epithelium [2]. Dozens of clinical cases of *C. fetus* causing infected aneurysms have been described, mostly of the abdominal aorta [3,4,5]. Furthermore, vascular prosthesis infections following arterial reconstructive surgery are rare but serious complications, when the infected graft must be removed. Among several replacement materials, biologic allografts have shown good results in terms of reinfection rate, durability, accessibility and surgical trauma [6,7].

Herein, we describe a unique case of *C. fetus* bacteremia related to vascular prosthesis and pseudoaneurysm infection, with a review of previously published cases related to cardiovascular prosthetic devices.

## 2. Materials and Methods

**Figure 1:**
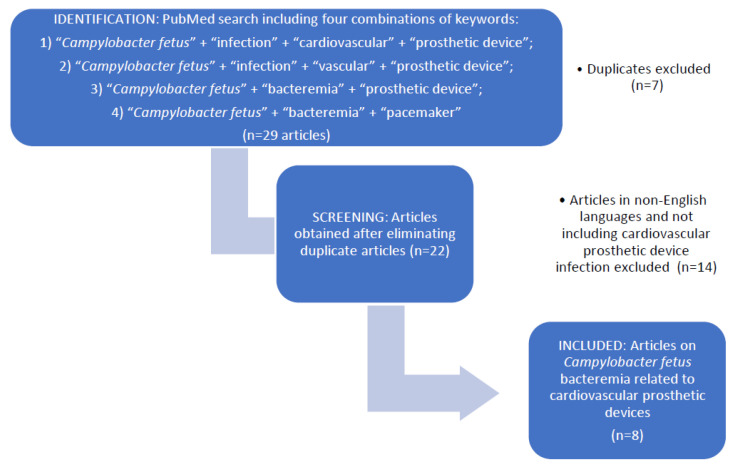
Flow diagram of studies included in the review.

A PubMed search for clinical reports of *Campylobacter fetus* bacteremia using the search keywords “*Campylobacter fetus*,” “bacteremia,” “vascular,” “cardiovascular,” “prosthetic device,” “pacemaker” and “infection” within the period from 1980 to 2022 was performed. Only full-text articles in English were included. Articles in other languages, as well as articles with abstracts but no full-texts available, were excluded from the review (Figure 1). Out of 22 articles published, only eight describing *C. fetus* bacteremia related to cardiovascular prosthetic devices were included in this review (Table 1) [8,9,10,11,12,13,14,15,16].

Blood cultures were incubated in an automated BacT/Alert system (bioMerieux, Marcy-l’Étoile, France). Identification of *C. fetus* was performed by a VITEK 2 (BioMerieux). Antibiotic susceptibility testing was performed on Mueller-Hinton agar with 5% sheep blood under microaerophilic conditions using commercial E-test strips for minimum inhibitory concentration (MIC) determination. MIC values were interpreted according to the breakpoints outlined in Version 9.0 of the European Committee on Antimicrobial Susceptibility Testing (EUCAST) Guidelines (www.eucast.org, accessed on 14 July 2022).

## 3. Results

### Case Report

In July 2019, a 67-year-old male was referred to the Dubrava University Hospital (Zagreb, Croatia) from another tertiary care hospital, where he had been admitted with a history of three days of fever >38 °C accompanied by general weakness and painful swelling of the right groin. He denied diarrhea, abdominal pain or any other gastrointestinal symptoms. Symptomatic treatment and empirical ciprofloxacin 400 mg intravenously (IV) q12h were administered, followed by co-amoxicillin 1.2 g IV q8h, without any improvement. After unsuccessful treatment, the patient was transferred to the Dubrava University Hospital, where he had been previously treated for aortoiliac occlusive disease on several occasions. In 2007, percutaneous transluminal angioplasty with stent placement and thrombectomy of the iliac and femoral arteries were performed. After multiple stent placements and thrombectomy procedures, his condition severely deteriorated in 2012, when a prosthetic aortobifemoral and popliteal bypass with polyester graft were placed. Besides his peripheral vascular disease, he had arterial hypertension, coronary artery disease with stent placement, an implantable cardioverter-defibrillator due to ventricular tachycardia, diabetes, chronic obstructive pulmonary disease and chronic renal insufficiency. The patient had no liver disease, malignancy, AIDS or other immunodeficiency.

He was a retired sailor who lived with his wife and had no pets or domestic animals. There was no history of recent travel abroad, drinking unpasteurized milk or eating undercooked meat. However, he grew vegetables for which he used sheep manure as fertilizer, provided by a neighbor who raised sheep.

On admission to the Dubrava University Hospital, his laboratory workup was significant for an elevated white blood cell count of 13.8 × 10^9^/L (reference range: 3.4–9.7) and a C-reactive protein level of 289.7 mg/L (reference range: <5). Other relevant laboratory workup included creatinine 249 µmol/L (reference range: 64–104), urea 27.9 mmol/L (reference range: 2.8–8.3) and thrombocytes 407 × 10^9^/L (reference range: 158–424). A repeated multi-slice computer tomography angiography (MSCTA) showed a pseudoaneurysm in the right groin measuring 84 × 76 mm. Two pairs of blood cultures were drawn before antimicrobial treatment with ciprofloxacin was initiated.

On the fourth day after admission, repair of the pseudoaneurysm, removal of the femoral part of the aortofemoral prosthesis in the right groin and biologic graft placement in the right groin were performed. A tissue sample of the pseudoaneurysm and a part of the removed vascular prosthesis were sent to the microbiology laboratory for culture and susceptibility testing. On the seventh hospital day, i.e., the third day post-surgery, growth was detected under anaerobic conditions at 37°C on anaerobic plates from blood cultures as well as tissue and prosthesis samples. Based on specific Gram stain morphology, the isolate was presumptively identified as *Campylobacter* spp. and a clinical microbiologist suggested a change in antimicrobial therapy from ciprofloxacin to intravenous meropenem 1 g IV q8h. The result yielded on a VITEK2 was *C. fetus* with 98% confidence, which was deemed by the software as an excellent identification. The isolate was found to be susceptible to amoxicillin (MIC 0.5 mg/L), imipenem (MIC 0.032 mg/L), meropenem (MIC 0.05 mg/L), gentamicin (MIC 0.38 mg/L), tetracycline (MIC 0.5 mg/L) and azithromycin (inferred from erythromycin disk diffusion) but resistant to ciprofloxacin (MIC 32 mg/L). After two weeks, the patient was sent back to the hospital he had been transferred from for continued antimicrobial treatment. Meropenem was administered for another two weeks, with a recommendation for de-escalation to oral amoxicillin-clavulanate for another two weeks after discharge. At subsequent visits, the patient appeared well and made a full recovery. The final follow-up was conducted by telephone a year after the procedure. The patient was well, with no signs of residual disease or relapse.

To our knowledge, there are eight previously published cases of *C. fetus* bacteremia due to infected cardiovascular prosthetic devices, summarized in Table 1.

These infections occurred in the presence of prosthetic heart valves [8,9,10,12,14,15,16], implantable cardioverter-defibrillators [11] and a permanent pacemaker [13]. However, none of them were related to an infected post-surgical vascular prosthesis and pseudoaneurysm as in our case.

## 4. Discussion

We report a rare case of *C. fetus* bacteremia related to an infected post-surgical vascular prosthesis and pseudoaneurysm with a review of previously published cases related to other cardiovascular prosthetic devices. It is well established that prosthetic material is the perfect ground for bacteria from the blood, which form a biofilm [17], as reported for *C. fetus* by Lynch et al. [15].

Our patient had several risk factors for *Campylobacter fetus* infection: he was an elderly individual with comorbidities, which possibly made him immunocompromised. It is unclear which segment of the immune system should be most compromised for infection to occur. Since *C. fetus* possesses a surface layer protein, which shields it from opsonizing antibodies and the complement, defects in the complement pathway may be conducive to infection [1,2]. Additionally, he may have been exposed to a high infective dose, considering that he had used sheep manure as fertilizer for his garden. It is well established that the primary reservoirs of *C. fetus* are cattle and sheep [1,2]. *C. fetus* primarily infects immunocompromised hosts and the elderly, although if the infective dose is high enough, it is known to infect healthy individuals, who are most often professionally exposed to animals, i.e., farmers and slaughterhouse workers [1,2]. The third risk factor that contributed to the development of his infected vascular prosthesis and pseudoaneurysm, in the context of what otherwise might have been transient bacteremia, was arterial damage and the presence of a foreign body: the vascular prosthesis.

With so few known cases of cardiovascular prosthetic device infections caused by *C. fetus*, it is difficult to provide a general recommendation for the best approach to treatment. The decision whether to remove or salvage the prosthesis should be made on a case-by-case basis. However, all the patients in Table 1 were somewhat clinically unstable, which in most instances prompted the authors to remove the prosthetic devices. In the case of our patient, there was clear indication for the repair of the pseudoaneurysm and removal of the partial vascular prosthesis because he exhibited signs of systemic infection, despite antimicrobial therapy. In addition, there was the imminent threat of pseudoaneurysm rupture and consequent lethal hemorrhage. We decided to repair the pseudoaneurysm and replace the infected part of the aortobifemoral prosthesis in the right groin with a biologic graft (made from bovine pericardium), due to its proven resistance to infection [18].

The choice and duration of antimicrobial therapy for *C. fetus* bacteremia related to cardiovascular devices are also subject to debate, owing to the small number of cases. However, in our patient, given the failure of primary treatment with ciprofloxacin and amoxicillin-clavulanate and his deteriorating condition, escalation to meropenem was the right choice while awaiting culture and susceptibility test results. This was confirmed when the isolate identification and its susceptibility became available. EUCAST only defines breakpoints for two *Campylobacter* spp.—*Campylobacter jejuni* and *Campylobacter coli*—and only for macrolides, fluoroquinolones and tetracyclines. Therefore, susceptibilities to amoxicillin, amoxicillin-clavulanate, imipenem, meropenem, tetracycline and gentamycin were inferred from the PK-PD (non-species related) EUCAST breakpoints [19]. Clinical & Laboratory Standards Institute (CLSI) has breakpoints for *Campylobacter* spp., but not for carbapenems [20]. However, European Food Safety Authority (EFSA) has an epidemiological cut-off (ECOFF) value for ertapenem, but only for *C. coli* and *C. jejuni* [21]. This particular strain of *C. fetus* was resistant to ciprofloxacin, which is more the rule than the exception. In 2020, the resistance of *Campylobacter* spp. to ciprofloxacin exceeded 70% in Croatia [22]. *Campylobacter jejuni* and *Campylobacter coli* are generally resistant to ceftriaxone, but *C. fetus* might be susceptible. Therefore, ceftriaxone therapy could have been considered, although in such an infection it should have been combined with an antimicrobial having an anaerobic spectrum, such as metronidazole. Although the strain was susceptible to macrolides in vitro, they are not the optimal choice for treating bacteremia. After the patient was stable, de-escalation to amoxicillin-clavulanate was appropriate. He received effective antibiotic therapy for a total of five weeks, which, in retrospect, proved to be sufficient for his complete recovery.

## 5. Conclusions

*Campylobacter fetus* has an affinity for the endothelium and must be considered as a potential pathogen in the presence of aneurysms, especially in conjunction with a vascular prosthesis. To our knowledge, this is the first report of a *C. fetus* infection of a vascular prosthesis causing a pseudoaneurysm. The surgical procedure comprised the resection of the infected part of the vascular prosthesis and its replacement with a biologic prosthesis. This procedure and concomitant antimicrobial therapy of four weeks of intravenous meropenem and two weeks of orally administered amoxicillin-clavulanate resulted in the resolution of the infection and the patient’s full recovery. Further studies involving a larger number of patients are needed to optimize treatment regimens in light of increasing antibiotic resistance.

## Figures and Tables

**Table 1:** *Campylobacter fetus* bacteremia related to cardiovascular prosthetic devices: literature review and present case.

	First Author [Ref.]/Country/ Publication yr.	Age (yr.)/ Sex	Type of Cardiovascular Prosthetic Device (Time from Implantation to Presentation)	Underlying Condition/Risk Factor	Type of Antimicrobial Therapy (Duration)	Removal of the Prosthesis, Type of Surgery	Outcome
1.	Caramelli [8]/Brasil/1988	48/F	Biologic prosthetic mitral valve (9 yr.)	None	PEN 24 IU/day + STR 600 mg/day followed by GEN 180 mg/day (42 days)	Yes, replaced by bovine prosthetic valve	Cured
2.	Farrugia [9]/UK /1994	76/F	Aortic Carpentier-Edwards valve (3 yr.)	Elderly	PEN 1.8 g/q6h + GEN 80 mg/q12h followed by AMX 1 g /q6h (22 days) + GEN 80 mg/q12h (15 days) CIP 500 mg/q12h orally	Yes, replaced with a porcine valve prosthesis	Cured (follow-up for 8 mo.)
3.	Peetermans [10]/Belgium/2000	61/M	Prosthetic aortic valve (25 yr.)	Elderly, gastric ulcer	ERY + GEN	No	Fatal
4.	Ahmar [11]/Australia/2008	77/M	Internal cardioverter defibrillator (5 yr.)	Hypertension, hypercholesterolemia, diabetes	FLX + CRO followed by MEM AMX 1 g/q8h (10 days)	Yes, ICD and lead were explanted without complication; a new ICD was inserted 1 mo. later	Without residual illness at the time of the implantation of the new ICD
5.	Haruyama [12]/Japan/2011	65/F	Prosthetic aortic valve (5 yr.)	Dental caries, habitual eating of raw meat	CFD 300 mg/day one wk. before admission First hospitalization Empirical: VAN (1 g/day) + GEN (120 mg/day) (5 days) Targeted: AMP 6 g/day + GEN 120 mg/day (2 wk.) followed by AMP 8 g/day (4 wk.) and a subsequent oral AMX 1 g/day (6 wk.) Second hospitalization (4 wk. after the first discharge) IMI 1.5 g/day + GEN 120 mg/day (4 wk.) followed by oral AMX 1 g/day (duration unknown)	No	Cured (follow-up for 3 yr.)
6.	Ledina [13]/Croatia/2012	72/M	Permanent pacemaker (2 wk.)	Myelodysplasia, impaired fasting glucose levels	VAN + NET; CIP + NET	Yes, reimplantation of a new pacemaker	Cured
7.	Reid [14]/USA/2016	Late 70s/M	Prosthetic aortic valve (4 yr.)	Travelled to southeast Asia and Europe in the 6 wk. prior to disease onset, eating steak tartare, visited dentist 4 wk. prior; prophylactic AMX at that time	CRO 2 g/q12h followed by MEM 2 g/q8h on day 3 of hospitalisation; MEM switched to ETP 1 g/day (completed a 6-wk. course).	Yes, valve replacement	Full recovery
8.	Lynch [15]/Ireland/2019 Lynch [16]/Ireland/2022	77/M	Prosthetic valve (unknown)	None	Unknown	Unknown	Recurrent endocarditis
9.	Present case	67/M	Prosthetic aortobifemoral and popliteal bypass with polyester graft (7 yr.)	Arterial hypertension, coronary artery disease with stent placement, ventricular tachycardia, an implantable cardioverter-defibrillator, diabetes, COPD, chronic renal insufficiency; he grew vegetables for which he used sheep manure as fertilizer	CIP 400 mg/q12h followed by AMC 1.2 g/q8h (prior to admission); CIP 400 mg/q12h (3 days) followed by MEM 1 g/q8h (4 wk.)	Yes, pseudoaneurysm repair, removal of the femoral part of the aortofemoral prosthesis and biologic graft placement (made from bovine pericardium)	Cured (follow-up for one yr)

COPD: chronic obstructive pulmonary disease, F: female, ICD: internal cardioverter defibrillator, g: gram; mg: milligram, mo.: month, q6h: every 6 h, q8h: every 8 h, q12h: every 12 h, UK: United Kingdom, USA: United States of America, wk.: week(s), yr.: year(s). AMC: Amoxicillin + clavulanic acid, AMP: ampicillin, AMX: amoxicillin, CFD: cefdinir, CIP: ciprofloxacin, CRO: ceftriaxone, ERY: erythromycin, ETP: ertapenem, FLX: flucloxacillin, GEN: gentamicin, IMI: imipenem-cilastatin, MEM: meropenem, NET: netilmicin, PEN: penicillin, STR: streptomycin, VAN: vancomycin.

## Data Availability

Not applicable.
